# Channel activity of mirror-image M2 proton channel of influenza A virus is blocked by achiral or chiral inhibitors

**DOI:** 10.1007/s13238-018-0536-5

**Published:** 2018-04-20

**Authors:** Qing-Yan Guo, Long-Hua Zhang, Chao Zuo, Dong-Liang Huang, Zhipeng A. Wang, Ji-Shen Zheng, Chang-Lin Tian

**Affiliations:** School of Life Sciences, University of Science and Technology of China, Hefei, 230027 China

**Dear Editor,**

It is known that most of lives on the earth compose of homo-chiral molecules of L-amino acids and D-ribose nucleic acids. However, little is known why and how the life’s chirality in such a way. Studies on an artificial mirror-image life could strengthen our understanding of the question about the origin of life on the Earth and even elsewhere in the universe. Especially studies on mirror-image life would also have a plenty of vast application prospects in materials, energy and pharmaceutical sciences (Bohannon, 2010). Recently, several significant preliminary explorations on soluble proteins have been conducted. For instance, the Kent’s group achieved the chemical synthesis of enantiomeric HIV-1 protease and found its mirror-image specificity on the peptide substrate (Milton et al., 1992). The d-DapA was synthesized to study chaperone-assisted protein folding problems (Weinstock et al., 2014). The mirror-image ASFV pol X polymerase was synthesized and performed the mirror-image genetic replication and transcription in the central dogma (Wang et al., 2016). A thermostable d-Dpo4 for mirror-image PCR was also successfully achieved (Xu et al., 2017). These pioneering works open a window for studies of mirror-image lives. However, the mirror-image hydrophobic membrane proteins and their functional property studies remain untouched. Similar as hydrophilic proteins, mirror-image studies of membrane proteins (e.g., ion channels) will provide more insights about mirror-image cells or lives.

Cell membrane is a protective hydrophobic barrier in a living cell, which prevents the free entry of extracellular substances and makes the intracellular environment relatively stable to guarantee the orderly operation of various intracellular biochemical reactions (Szostak et al., 2001). To produce artificial mirror-image cells, a key challenge lies in the construction of ion channels with selective ion permeability. Our recent developments on chemical synthesis of membrane proteins can readily achieve the preparation of small- to medium-sized membrane proteins using the removable backbone modification (RBM) strategy (Zheng et al., 2014). Therefore, we implement the chemical synthesis of D-M2 protein and channel reconstitution for a model system to study the ion permeability of the mirror-image ion channels. The L-/D-M2 ion channels were chosen as the model system for two reasons. First, proton gradients mediated by ion channel across membranes may play a key role in the origin of life at the very specific alkaline environment (Lane and Martin, 2012). Secondly, the virus fusion and uncoating of influenza A virus capsid is governed by the M2 ion channel.

Herein, we report the first chemically synthesized D-amino acid ion channel with proper conductance function. The mirror-image M2 proton channel (D-M2) of influenza A virus was prepared by the combination of two techniques of RBM strategy and peptide-hydrazide-based native chemical ligation. The consequent electrophysiological experiments showed that the mirror-image D-M2 channel acted as a proton-conducting channel, similar as the native L-M2 channel. Furthermore, the amantadine-based anti-influenza drugs achiral amantadine and chiral R-rimantadine showed inhibitory effect on D-M2 channel. This study would provide preliminary but valuable insights into the action that *in vitro* interfering molecules target to mirror-image life forms.

To synthesize D-M2 protein containing 97 D-amino acids, chemical synthesis of two peptide segments D-M2[1–49, 4Arg-Tag]-NHNH_2_ 1 and D-M2[50–97] 2, was conducted, similar to the previously reported procedure for L-M2 (Zheng et al., 2014). An Arg4-tagged backbone modification group was incorporated into the transmembrane domain containing peptide 1 at Gly34 to improve the solubility of 1. Peptides 1 and 2 were successfully prepared by 9-fluorenylmethoxy-carbonyl solid-phase peptide synthesis (Fmoc SPPS) with the isolated yield of 16% and 21%, respectively. The ligation of 1 and 2 was performed using the protocol of hydrazide based native chemical ligation (Fang et al., 2011; Pan et al., 2016). The ligation product was purified using the reversed-phase high-performance liquid chromatography (RP-HPLC) to give D-M2[1–97, 4Arg-Tag] 3 with 30% yield. The peptide 3 was treated with TFA cocktails for 5 h to remove the backbone modification groups. The final product D-M2 4 was obtained in 19% yield by RP-HPLC purification.

The product was verified by electrospray ionization mass spectrometry (ESI-MS) (Fig. 1C). The sodium dodecyl sulfate-polyacrylamide gel electrophoresis (SDS-PAGE) analysis also confirmed the purity and molecular weight of 4 (Fig. 1D). Note that the L-M2 protein was synthesized by the second-generation RBM strategy (Tang et al., 2017). Compared to the previously reported strategy in 2014 (Zheng et al., 2014), the second-generation RBM strategy is that it can introduce the solubilizing RBM tag at any primary amino acid and facilitate the synthesis of L-M2. The synthetic L- and D-M2 proteins were analyzed using circular dichroism (CD) spectroscopy. The results showed that the CD spectrum of D-type M2 protein has the maximum absorption at 208 and 222 nm, which is chiral symmetric with L-M2 protein. The result is consistent with the previous results of enantiomers (Milton et al., 1992; Wang et al., 2016). These CD spectra indicate chemically synthesized D- and L-M2 both correctly folded with mirror-symmetrical structures.

**Figure 1:**
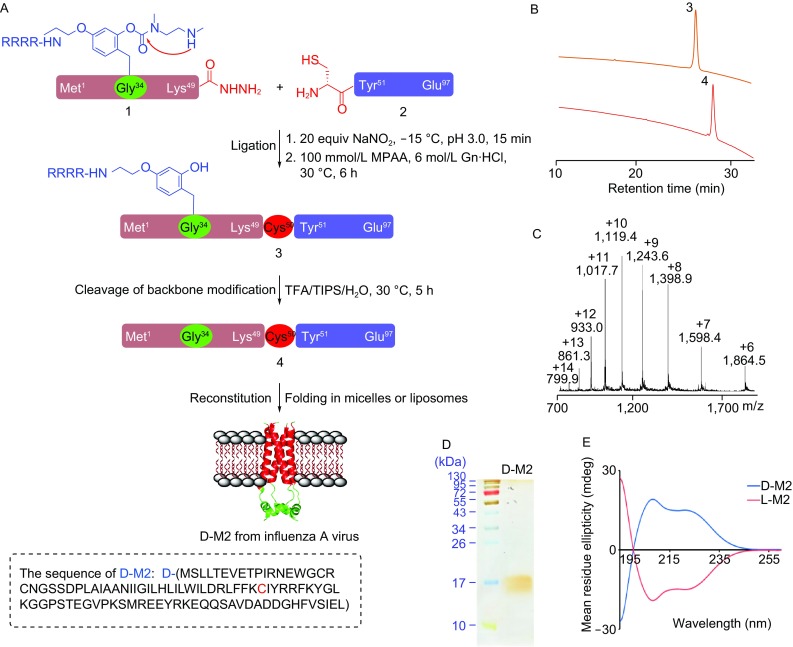
**Peptide hydrazide-based chemical synthesis of D-M2 protein using the RBM strategy and protein characterizations**. (A) General synthetic route; (B) HPLC traces of purified RBM-tagged D-M2 and native D-M2; (C) ESI-MS of D-M2; (D) SDS-PAGE and silver staining analysis of D-M2; (E) Circular dichroism spectra of the folded L-M2 and D-M2. ~5 μmol/L of L-M2, D-M2 in 20 mmol/L Tris, 50 mmol/L OG, 50 mmol/L NaCl (pH 7.3) is measured respectively in a 1 mm quartz cell

It is noted that the chemically synthesized D-M2 could be used for new antiviral drug development. The D-protein based mirror-image phage display technology may represent a very promising way to obtain D-peptide anti-influenza drugs with high stability and low immunogenicity (Schumacher et al., 1996; Chang et al., 2015).

With D-M2 protein in hands, the ion conductance properties of D-M2 channels were tested and compared with that of L-M2 channels. The L- and D- M2 proteins were respectively incorporated into lipid bilayers which are composed of POPC/POPG (3/1; POPC: 1-palmitoyl-2-oleoylsn-glycero-3-phosphocholine; POPG: 1-palmitoyl-2-oleoyl-snglycero-3-phosphor-(1′-rac-glycerol)). The mixture was dialyzed against Tris buffer (20 mmol/L Tris, 200 mmol/L NaCl, pH 8.0) for four days at 4 °C to completely remove the detergent. The single channel conductivity experiments were carried out using the instrument of Ionovation Compact (Osnabruck, Germany). Single-channel activities were examined using a ramp voltage protocol from −100 to 100 mV, or a series of step protocols to the given applied voltages (−60, −40, −20, 0, 20, 40 or 60 mV). As shown in Figure 2A and 2B, both ramp and step protocols showed that the channel current was not obvious at relatively low potentials and the typical burst current appeared at high potentials (−60, −40, 40, 60 mV or even higher) and the maximal amplitude of burst currents increased with the increase of potential. Meanwhile, control experiments without addition of D-M2 or L-M2 did not produce any current under the same conditions (data not shown). It is concluded that the incorporation of D-M2 protein can result in typical burst currents at both positive and negative potentials (Fig. 2B), similar to that of L-M2 (Fig. 2A). The orientation of burst currents showed flow of positive ions in both D-M2 and L-M2. To further compare the gating characteristics of L- and D-type M2 channels, we tested their burst rate at +60 mV. We set half average currents as open threshold, used a long recording trace of 15 s or a group of multiple traces more than 15 s, and calculated the open probability of L- and D-type M2 channels in planar lipid bilayers. 19 times L-M2 and 37 times D-M2 protein incorporations were examined. The statistics showed that both enantiomeric channels almost have the similar open probability (47.08% ± 13.38 % of D-M2 vs. 48.41% ± 19.35 % of L-M2) (Fig. 2C). Those results indicated that the reconstituted D-M2 channels can form functional homo-tetrameric H^+^ ion channels to produce single-channel activity, similar as our previous report of L-M2 channels (Zheng et al., 2014). We observed the ion permeation properties of the synthetic mirror-image D-M2 channel for the first time.

**Figure 2:**
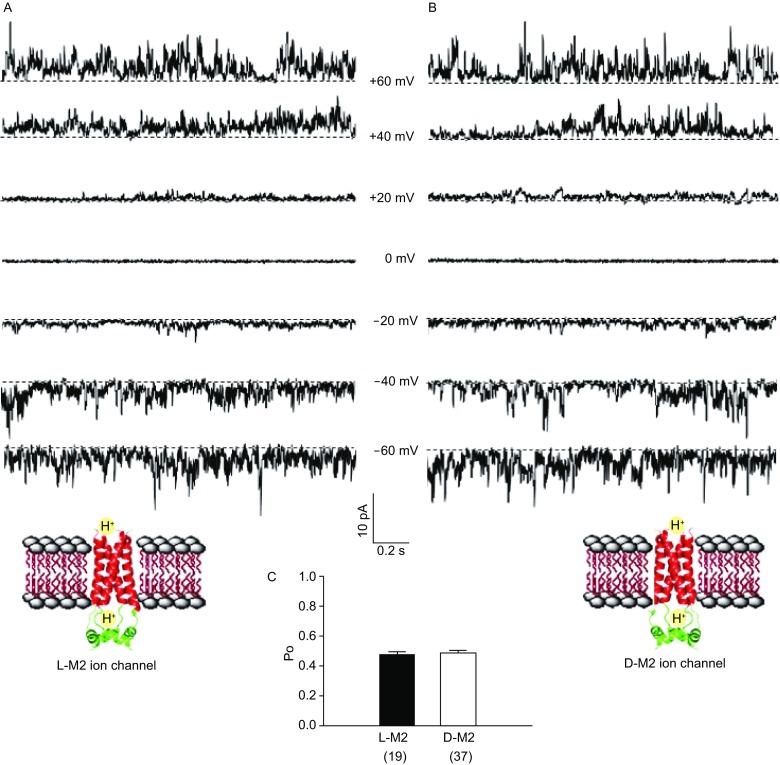

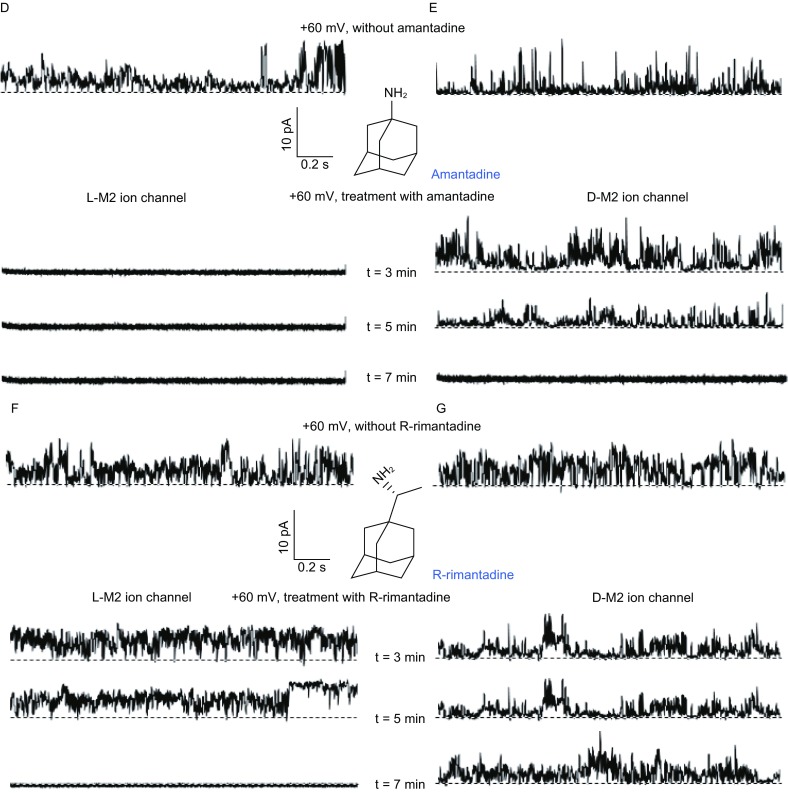
**Single-channel currents of chemical synthetic**. (A) L-M2 and (B) D-M2 ion channels after reconstitution in POPC/POPG (3:1) lipid vesicles; (C) the open probability of L-/D- M2 channels at +60 mV. The traces were recorded at various membrane potentials; the inhibitory effect of achiral inhibitor amantadine (20 μmol/L) on (D) L-M2 and (E) D-M2; chiral inhibitor R-rimantadine (20 μmol/L) on (F) L-M2 and (G) D-M2. Dotted line: closure

Furthermore, we investigated the achiral or chiral compounds that can regulate the conductance activity of L-/D-M2 channels. The inhibitory effect of achiral amantadine was first tested, which was reported to be a canonical channel blocker of L-M2 proton channel (Wang et al., 1993). In brief, amantadine was dissolved into DMSO (3 mmol/L). After the incorporation of D-M2 into lipid bilayers, we recorded the single-channel currents of a D-M2 channel before and after the application of amantadine (20 μmol/L) at +60 mV. We also tested the inhibitory effect of amantadine on L-M2 channel as a reference. As shown in Figure 2D, 20 μmol/L of non-chiral amantadine showed a complete inhibitory effect on L-M2 channel, blocked L-M2 channel activity within 3 min. It was found that amantadine can also inhibit D-M2 channels but take longer time, which attenuated D-M2 currents in 5 min and completely blocked the channel in 7 min (Fig. 2E). The relatively longer time for D-M2 channel inhibition might be caused by the compatibility between D-M2 proteins with surrounding chiral lipids.

Finally, the inhibitory effects of R-rimantadine (chiral inhibitor) on L- and D-M2 channels were examined and the results were shown in Fig. 2F and 2G. At a concentration of 20 μmol/L, R-rimantadine can also block L-M2 channel within 7 min. However, R-rimantadine did not block D-M2 channel even up to 15 min. We then stirred and waited for 10 min, and the channel activity of D-M2 was blocked at +60 mV (Supplementary Material). Burst activities came back at higher potentials (lower than −80 mV or higher than +100 mV), which suggested that the potential competition between the adding potentials and applied inhibitors revealed an incomplete blockage. These results demonstrated that thermal dynamically, there is no obvious chiral selectivity in the inhibition of M2 ion channel enantiomers, while kinetically, there is some chiral preference or bias for the inhibitors of the channel enantiomers. These observations also indicated that the ion channel activity inhibition was mainly determined by the blocking effect of the achiral amantadine moiety, which is consistent with previous reports (Drakopoulos et al., 2017).

In summary, we totally chemically synthesized D-M2 proteins using the RBM strategy and peptide hydrazide-based native chemical ligation. The chemically synthesized D-M2 proteins were reconstituted in lipid bilayers. The artificial D-M2 channel possesses the proton conductivity which can be blocked by amantadine-based inhibitors. We anticipate that the further investigation of the chemically synthesized other D-type transmembrane ion channels (such as proton, sodium (Nav), potassium (Kv) or calcium (Cav) channels), in combination with other looking-glass versions of biochemical processes, could enable the reconstutition of mirror-image cells as powerful tools for materials science and pharmaceutical research.

## Electronic supplementary material

Below is the link to the electronic supplementary material.

Supplementary material 1 (PDF 1036 kb)
