# Supplementary material for: Channel activity of mirror-image M2 proton channel of influenza A virus is blocked by achiral or chiral inhibitors
Source: Protein Cell. 2018 Apr 20;10(3):211–6. doi: 10.1007/s13238-018-0536-5 (PMC6338619; doi:10.1007/s13238-018-0536-5)
Supplement: Supplementary file 1 — Supplementary material 1 (PDF 1036 kb) [file 13238_2018_536_MOESM1_ESM.pdf]

Supplementary Information

Table of Contents

General experimental section••S2

Fig. S1. Synthesis of D-M2[1−49, 4Arg-Tag]-NHNH$_2$ 1•••••••••••••••••••••••••••••••S5

Fig. S2. Synthesis of D-M2[50-97] 2 •••S6

Fig. S3. RP-HPLC traces for ligation of 3••S7

Fig. S4. ESI-MS data of 3' and 3•••S8

Fig. S5. Single-channel currents of chemical synthetic L-M2 and D-M2 ion channels after reconstitution in POPC/POPG (3:1) lipid vesicles•••••••••••••••••••S10

Fig. S6. The inhibitory effect of R-rimantadine on D-M2•••••••••••••••••••••••••S11

EXPERIMENTAL SECTION

Peptide Synthesis[1]. The peptides D-M2[1-49] and D-M2[50-97] were synthesized using a C S Bio Co. automated synthesizer CS136XT with a scale of 0.2 mmol. The general protocols as follow: 2× DMF, 30 s; 1× 20% (vol/vol) piperidine in DMF, 5 min; 1× 20% (vol/vol) piperidine in DMF, 15 min; 2× DMF, 30 s; 2× DCM, 30 s; 3× DMF, 30 s; amino acid activation by HBTU or HCTU in the presence of DIEA in DMF for ~10 s; add AA solution (2 equiv) to the resin, and couple 1 h; 2× DMF, 30 s; 1× DCM, 30 s. a double coupling strategy is needed for sterically hindered amino acid derivatives (e.g., Fmoc-Ile-OH, Fmoc-Val-OH, Fmoc-Asn(Trt)-OH, Fmoc-Gln(Trt)-OH, Fmoc-Thr(tBu)-OH, Fmoc-Arg(Pbf)-OH and the amino acids to be coupled right after a Pro residue), especially when the assembled peptide is longer than 20 amino acids. The Gly34 was manually coupled as described in our previous work. When the assembly of peptide was complete, peptides were treated with TFA cocktails (82.5% TFA, 5% H_2O, 5% phenol, 5% thioanisole, 2.5% 1,2-ethanedithiol (EDT)), and purified by reversed-phase HPLC (CH_3CN-H_2O, 0.1% TFA). Each peptide was confirmed by electrospray ionization mass spectrometry (ESI-MS).

Chemical Ligation of D-M2 by Peptide Hydrazides[2]. 6.5 mg of peptide hydrazide D-M2[1-49, 4Arg-Tag]-$NHNH_2$ **1** was dissolved into 0.4 mL of phosphate solution containing 6 M Gn•HCl (pH 3.0-3.1) in a 2-ml Eppendorf tube. The reaction tube was placed in a -15 ^{o}C ice-salt bath. The cooled solution was gently agitated by magnetic stirring and 40 μL of $NaNO_2$ (0.5 M, 15 equiv) was pipetted. The solution was gently agitated for 15 min at -15 ^{o}C to oxidize the peptide hydrazide to azide. Subsequently, a phosphate buffer containing MPAA (4-mercaptophenylacetic acid, ~100 equiv) and 8.0 mg of D-M2[50-97] **2** peptide was added to the reaction mixture to convert the peptide azide to the thioester under the ice-salt bath. The reaction system was warmed to room temperature for the next ligation at pH 6.5-6.8. The ligation mixture was stirred for 3 h and then raised the pH to 7.4-7.5 to improve the intramolecular cyclization. The reaction was completed overnight at pH 7.2. The ligation was monitored by analytical HPLC and ESI-MS. The ligation mixture was subsequently reduced with 0.4 mL of 0.2 M TCEP solution for 30 min. After ligation completed, 0.4 mL of 0.2 M neutral TCEP solution was added for 20 min to reduce the ligation

system. The purified product D-M2[1-97,4Arg-Tag] **3** (3.7 mg, 30 % isolated yield) was obtained after preparative RP-HPLC purification.

The Removal of N-backbone Solubilizing Modification. The 4Arg-Tag was cleaved by TFA cocktails (82.5% TFA, 5% H_2O, 5% phenol, 5% thioanisole, 2.5% EDT). 3.5 mg of D-M2 protein was dissolved in 2 mL TFA cocktails and treated with 5 h. After that, TFA was concentrated by N_2 blowing and precipitated with Et_2O. The final product D-M2 was identified by ESI-MS (Calcd mass: 11183.6 Da and observed mass: 11183.6±0.8 Da) and purified by a further RP-HPLC purification in 19 % yield.

Preparation of Lipid Mixture for Bilayer. All lipids used were obtained from Avanti Polar Lipids (Alabaster, AL, USA). The POPC (1-palmitoyl-2-oleoyl-sn-glycero- 3-phosphocholine) and POPG (1-palmitoyl-2-oleoyl-sn-glycero-3- phosphor-(1'-rac-glycerol) (sodium salt) were stored at -80 °C, it should be recovered to room temperature before weigh. We weighed 10 mg lipid (POPC 7.5mg; POPG 2.5 mg) in two clean glass tubes and dissolved the lipids with 750 μL and 250 μL chloroform respectively. Then, two lipids were mixed, uniformly dispersed using pipettes and divided into to 20 tube subpackages. All the above operations were carried out on ice. The lipid solution was dried with N_2 for removal of chloroform and freeze-dried over 24 hours.

Protein Reconstitution in Unilamellar Liposome Vesicles. The chemical synthesized protein powder was centrifuged at 14,000 rpm for 10 minutes to recover the sample that sticks to the tube wall. Reconstitution buffer containing 0.1% SDS, 20 mM Tris, 200 mM NaCl, pH 8.0 was added into the tube. Sample was completely dissolved and uniformly dispersed using pipettes. The lipid mixture containing POPC and POPG were well dispersed in reconstitution buffer through 5-7 freeze-thawing cycles in liquid nitrogen and ultrasound for 5 min at room temperature. Then the protein solution and the lipid solution were mixed in reconstitution buffer with a molar ratio of 1/600 (protein/lipid) to reach a final concentration of the protein at 10~15 μg/ml. The mixture was rotated at room temperature for 1 hour and dialyzed for 4 days at 4 °C against Tris buffer (20 mM Tris, 200 mM NaCl, pH 8.0) for complete removal of the detergent. To prepare unilamellar liposome vesicles, samples were extruded 20 passes through a 0.1 μm polycarbonate membrane by the Avanti Mini-Extruder (Alabaster, AL). Final concentration of the proteoliposomes were ~15 μg/ml.

Single Channel Conductance Measurement in Planar Lipid Bilayer. Channel conductance measurements in planar lipid bilayer were conducted using Ionovation Compact (Osnabrück, Germany). Two polycarbonate compartments were separated by a TEFLON foil with 25 μm thickness and 50-100 μm aperture diameter and two Ag/AgCl electrodes immersed in each compartment. The pre-mixed lipid (POPC/POPG= 3/1) was dissolved by n-decane in the concentration of 20 mg/ml, then it was employed to paint the aperture. Planar lipid bilayer formation was monitored optically or by capacitance measurements. It will be suitable if the capacitance was among 45-100 pF, which will be easier for liposome fusion, so the protein can contact the planar lipid bilayer as well as formation of a channel to conduct the proton transport. After successful formation of a stable bilayer in the aperture, proteoliposomes (protein sample in POPC/POPG) were added to the cis chamber next to the bilayer. Fusion of protein and planar lipid bilayer was detected through observation of channel conductance, which was a signal to recording if the conductance was above 70 pS. Working solution (5 mM HEPES, 150 mM KCl, pH 8.0) were present in both cis- and trans- chambers in volume of 1.5 mL.

Ramp (from -100mV to +100mV for 10s) or step (to +60mV/-60mV at 20mV increasement, or to +120mV/-120mV at 10mV increasement) voltage protocols were applied across the bilayer to measure the channel conductance using an EPC-10 amplifier (HEKA Elektronik). Currents were measured with a 2 kHz low-pass filter at 10 kHz sampling rate. Data was analyzed using the pCLAMP 10.0 software (Axon Instruments). The channel activity (open probability) was measured in a 15s trace or a group of multiple traces combined to reach 15s.

Amantadine-based Inhibitors for M2 ion channels. The blocking effect of amantadine-based inhibitors (J & K scientific Ltd.) was performed as described by previous work.[1,3] These inhibitors (achiral amantadine and chiral R-rimantadine) were dissolved in DMSO to a stock concentration of 3mM and added to both cis- and trans-chambers to the final concentration of 20 μM. The effects of amantadine and R-rimantadine on D-M2 channels were performed.[3] We also tested the inhibitory effect of amantadine on L-M2 channel as a positive control.

References

1. J. S. Zheng, M. Yu, Y. K. Qi, S. Tang, F. Shen, Z. P. Wang, L. Xiao, L. Zhang, C. L. Tian, L. Liu, Expedient total synthesis of small to medium-sized membrane proteins via Fmoc chemistry. *J. Am. Chem. Soc.* **2014**, *136*, 3695-3704.
2. Fang, G. M.; Li, Y. M. S., F.; Huang, Y. C.; Li, J. B.; Lin, Y.; Cui, H. K.; Liu, L. *Angew. Chem. Int. Ed.* **2011**, *50*, 7645.
3. A. Drakopoulos, C. Tzitzoglaki, C. Ma, K. Freudenberger, A. Hoffmann, Y. Hu, G. Gauglitz, M. Schmidtke, J. Wang, A. Kolocouris, *ACS Med. Chem. Lett.* **2017**, *8*, 145-150.

SUPPLEMENTARY FIGURES

(A)

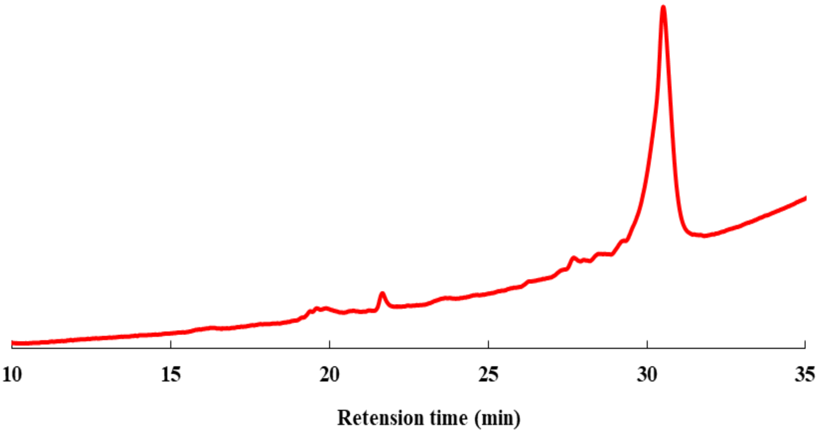

(B)

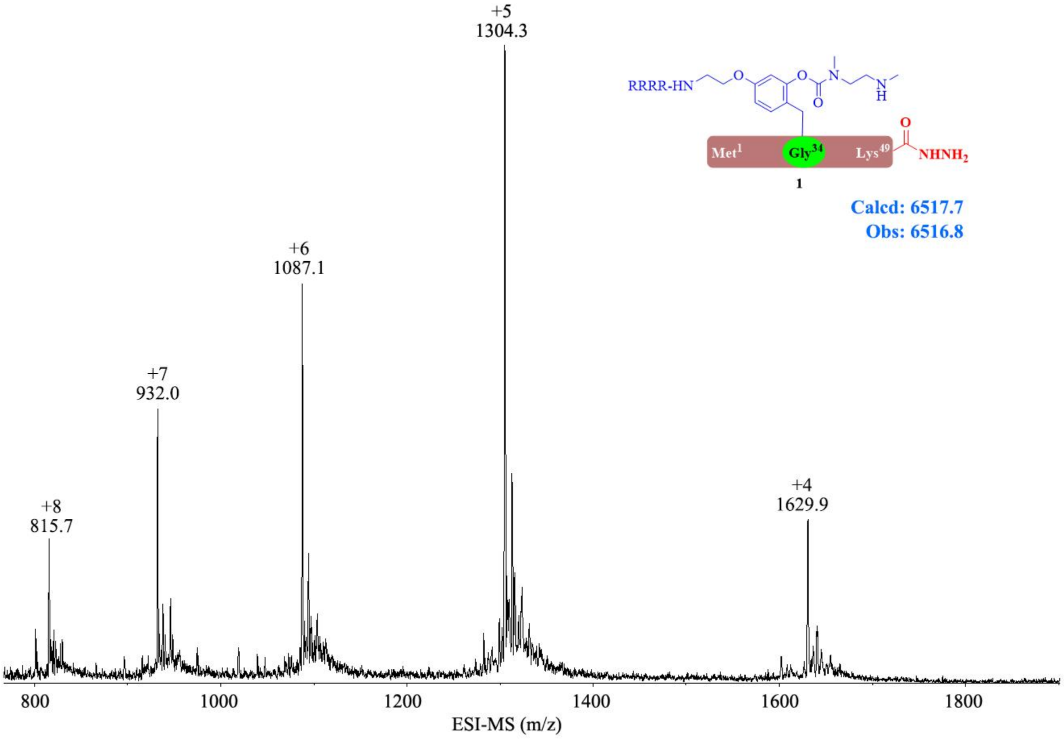

Fig. S1. Synthesis of D-M2[1−49, 4Arg-Tag]-$NHNH_2$ 1. (A) RP-HPLC analysis of the purified peptide **1** running on a C4-column (4.6×150 mm) at 56 °C. The linear gradient for analytical HPLC: 15% buffer B in buffer A to 75% B in A over 30 min. (B) ESI-MS of **1**.

(A)

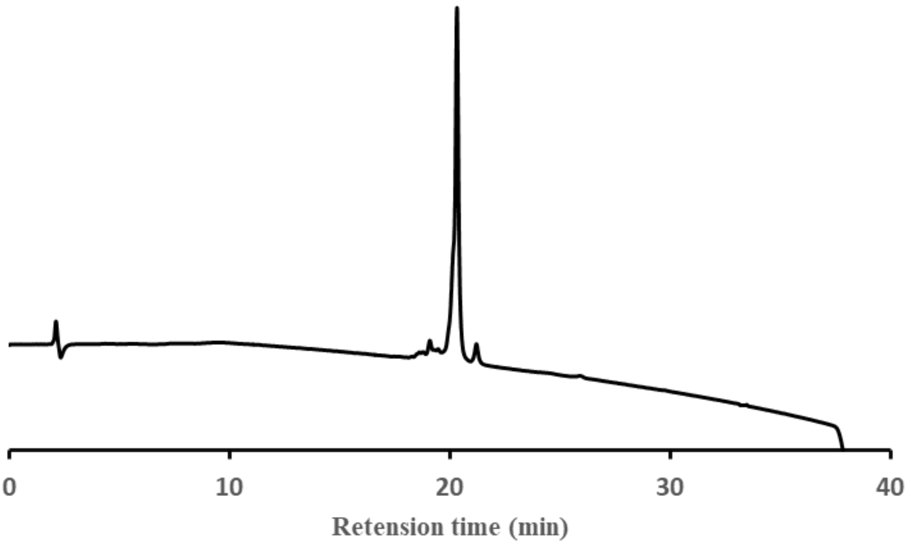

(B)

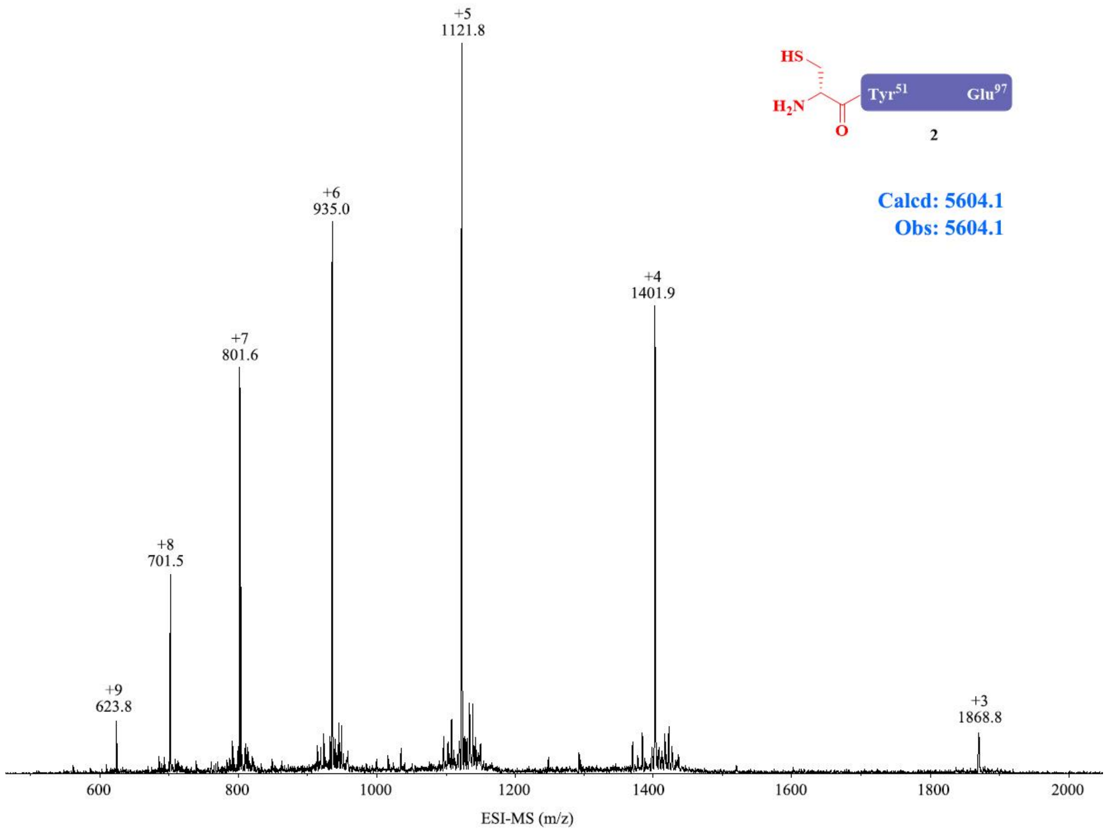

Fig. S2. Synthesis of D-M2[50-97] 2. (A) RP-HPLC analysis of the purified peptide **2** running on a C4-column (4.6×150 mm) at 56 °C. The linear gradient for analytical HPLC: 5% buffer B in buffer A to 65% B in A over 30 min. (B) ESI-MS of **2**.

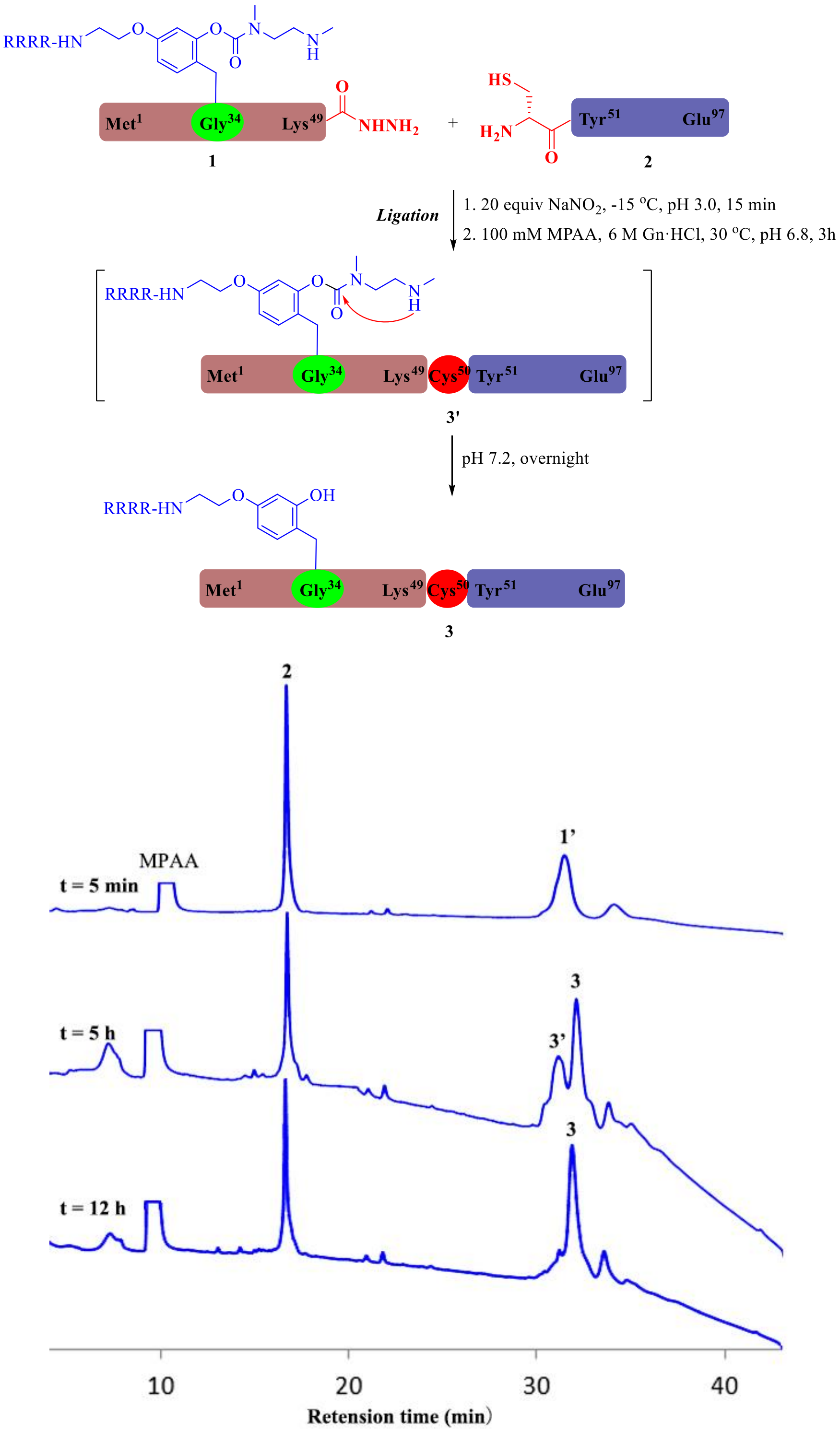

Fig. S3. RP-HPLC traces for the native chemical ligation of 3. RP-HPLC analysis was performed on a C4-column (4.6×150 mm) at 56 °C. The linear gradient for analytical HPLC: 15% buffer B in buffer A to 75% B in A over 35 min.

(A)

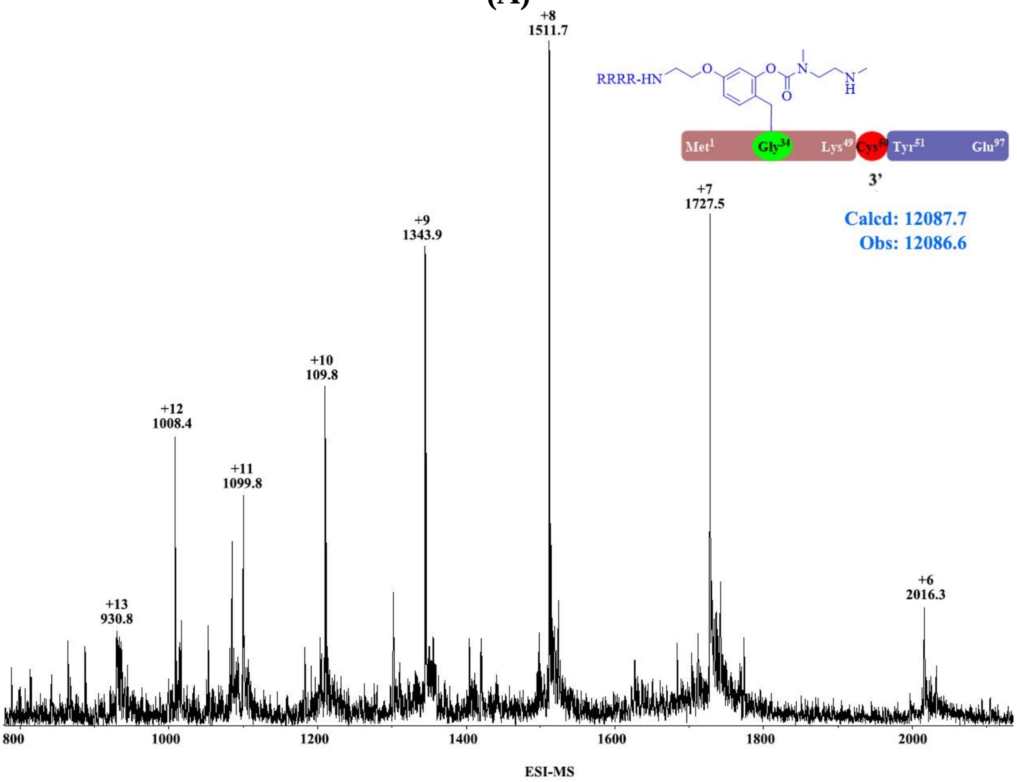

(B)

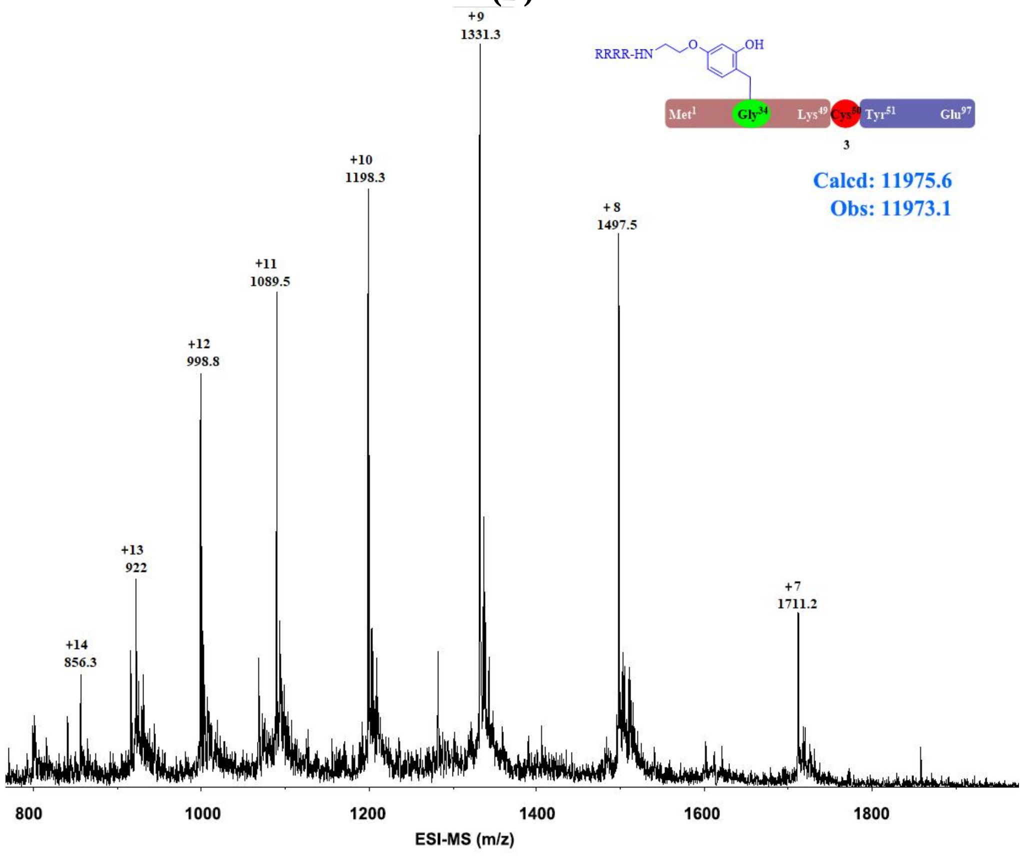

Fig. S4. ESI-MS data of (A) **3’ and** (B) **3.**

(A)

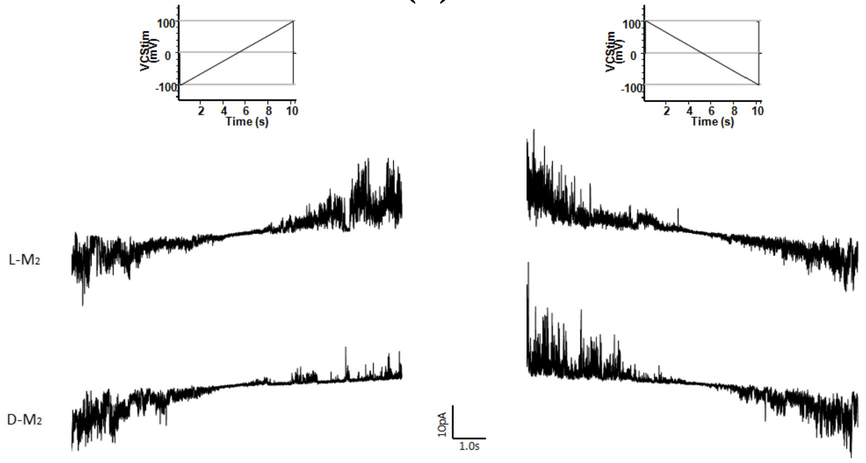

(B)

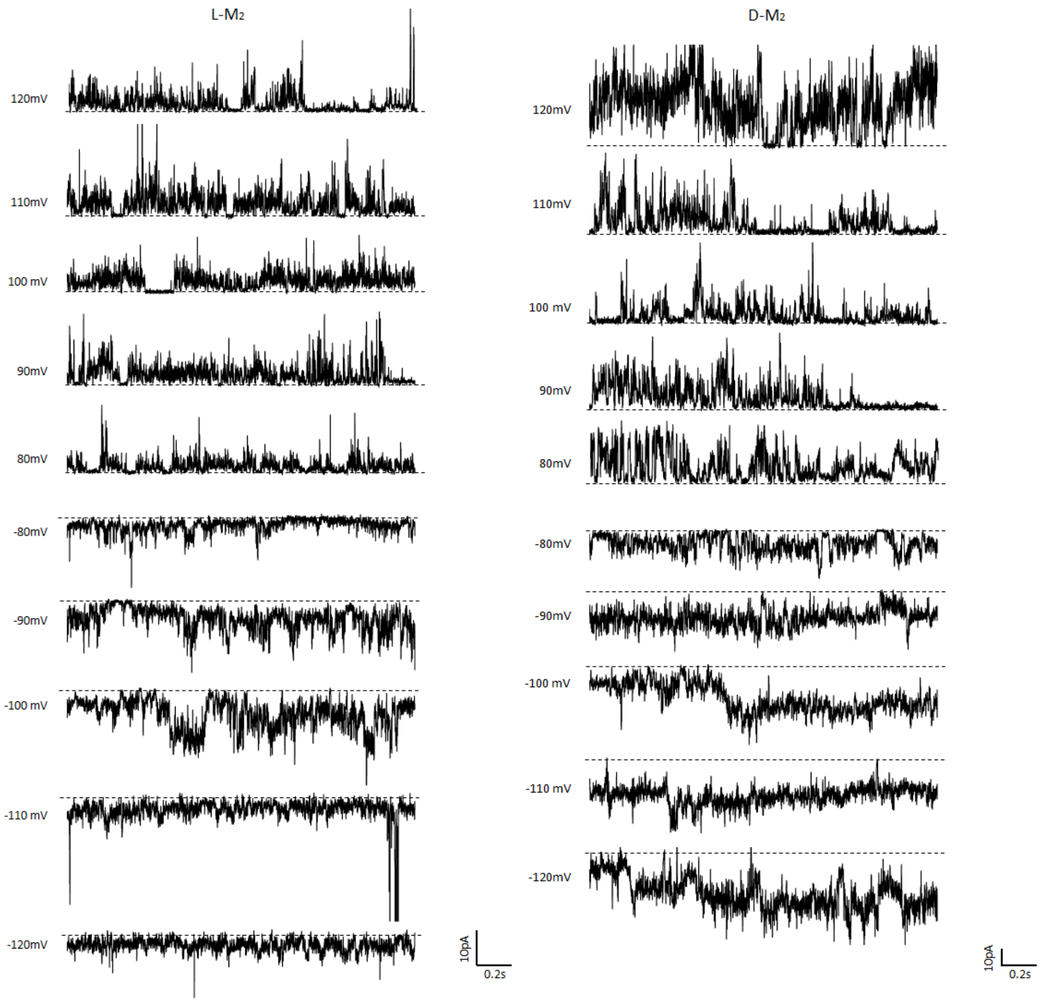

Fig. S5. Single-channel currents of chemical synthetic L-M2 and D-M2 in planar lipid bilayer. (A) The ramp protocol from -100 mV to +100 mV for 10 s (up) triggered continuous burst currents. Both of L-M2 (middle) and D-M2 (bottom) showed obvious increase in single channel currents along with the increase of voltages. (B)Single-channel currents of L-M2 and D-M2 under high voltages. Both L-M2 (left) and D-M2 (right) in high voltage showed similar burst characteristics.

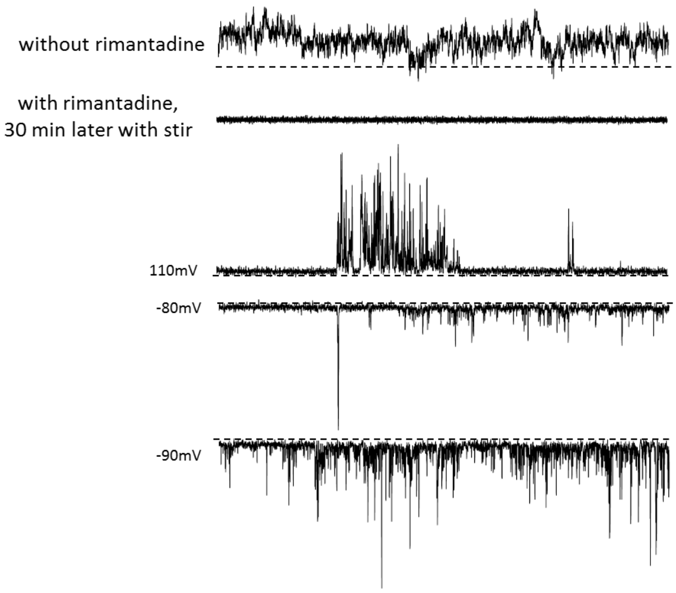

Fig. S6. The inhibitory effect of R-rimantadine on D-M2. All of these traces are from D-M2 ion channels recorded at +60 mV. R-rimantadine at a final concentration of 20 μM didn't shown obvious inhibitory effect until we wait up to 30 min (including stirred for 10 min). The inhibitory effect was reversed once we used high voltage step protocols.
